# Quantity judgments in the context of risk/reward decision making in striped field mice: first “count,” then hunt

**DOI:** 10.3389/fpsyg.2013.00053

**Published:** 2013-02-13

**Authors:** Sofia Panteleeva, Zhanna Reznikova, Olga Vygonyailova

**Affiliations:** ^1^Institute of Systematics and Ecology of Animals, Russian Academy of ScienceNovosibirsk, Russia; ^2^Novosibirsk State UniversityNovosibirsk, Russia

**Keywords:** quantity judgments, cognition, behavioral ecology, rodents, ants, risky hunting, feeding patches, decision making

## Abstract

We simulated the situation of risky hunting in the striped field mouse *Apodemus agrarius* in order to examine whether these animals are able to make a choice between small and large quantities of live prey (ants). In the first (preliminary) experiment we investigated to what extent mice were interested in ants as a live prey and how their hunting activity depended on the quantity of these edible but rather aggressive insects. We placed mice one by one into arenas together with ant groups of different quantities, from 10 to 60. Surprisingly, animals, both wild-caught and laboratory-reared, displayed rather skilled predatory attacks: mice killed and ate from 0.37 ± 003 to 4 ± 0.5 ants per minute. However, there was a threshold number of ants in the arenas when rodents expressed signs of discomfort and started to panic, likely because ants bit them. This threshold corresponds to the dynamic density (about 400 individuals per m^2^ per min) in the vicinity of anthills and ants' routes in natural environment. In the second experiment mice had to choose between different quantities of ants placed in two transparent tunnels. Ants here served both as food items and as a source of danger. As far as we know, this is the first experimental paradigm based on evaluation of quantity judgments in the context of risk/reward decision making where the animals face a trade-off between the hedonistic value of the prey and the danger it presents. We found that when mice have to choose between 5 vs. 15, 5 vs. 30, and 10 vs. 30 ants, they always tend to prefer the smaller quantity, thus displaying the capacity for distinguishing more from less in order to ensure comfortable hunting. The results of this study are ecologically relevant as they reflect situations and challenges faced by free-living small rodents.

## Introduction

Recent behavioral studies have given rise to a growing body of evidence that members of many species, from insects, fish and salamanders to rodents, dogs, cats, horses, dolphins, elephants, and primates, can judge about proportions and numbers of things, sounds, time intervals, smells, and so on. In this field of experimental animal cognition different levels of numerical competence have been revealed, from the ability to discriminate between clearly distinct quantities (relative numerousness judgments) to exact “counting” and arithmetic operations (see: Reznikova and Ryabko, 2011, for a detailed review). In nature, being able to perceive quantities is helpful in many situations, such as tracking predators, selecting the best foraging grounds or the best chance to mate. For example, beetles *Tenebrio molitor* (Carazo et al., 2009, 2012) and meadow voles *Microtus pennsylvanicus* (Ferkin et al., 2005) demonstrated the ability to discriminate between “more or less smells” left by competitive males within the limit of four; honey bees can “count” landmarks within the same limit (Chittka and Geiger, 1995; Dacke and Srinivasan, 2008); female lions can judge about the number of possible intruders by “counting” unfamiliar roars also within the limit of four (McComb et al., 1994); spotted hyenas react with increasing vigilance to calls produced by one, two and three unknown intruders (Benson-Amram et al., 2011); fish use number estimation in order to join a greater shoal (Agrillo et al., 2008, 2011; Gòmez-Laplaza and Gerlai, 2011); North Island robins New Zeland robins (*Petroica australis*) use quantity judgments when retrieving and pilfering cashed food (Hunt et al., 2008; Armstrong et al., 2012); and ants of several species are able to estimate numbers of encounters with members of other colonies on their feeding territories (Reznikova, 1999; Gordon, 2010).

Different kinds of cognitive processes, including numerical discrimination, can be understood in terms of the ways in which species are cognitively adapted to their different ecological niches. Questions remain regarding the taxonomy of the development and organization of numerical information, and its relationship to other domains in human and non-human minds (Davis and Pérusse, 1988; Beran, 2008, 2012; Cantlon, 2012). Experiments on ants (Reznikova, 2007, 2008; Reznikova and Ryabko, 2011) and newly hatched domestic chicks that displayed the ability to perform very simple arithmetic operations (Vallortigara et al., 2010) enable researchers to appreciate core components of animals' numerical cognition. It is likely that some non-human animals possess a higher potential in numerical abilities than we had previously assumed. We are still far from understanding how these capacities evolved and to what extent they are adaptive, and more comparative studies in the context of animals' day-to-day ecological problems are needed.

In this study we focused on the capacity of small rodents for choosing between small and large quantities of live prey, both edible and dangerous, in simulated feeding patches. We simulated a paradoxical natural situation where animals could prefer to “go for less” instead of “going for more,” as is typically the case with spontaneous choice tasks. In behavioral ecology, theories of optimal foraging (MacArthur and Pianka, 1966; Pyke et al., 1977; Stephens and Krebs, 1986) predict that animals “go for more,” because they evolve foraging strategies that maximize their net energy gain when foraging. The ability to distinguish between quantities and to choose the larger one may be widespread in the animal kingdom. In experiments many species demonstrated discrimination between different quantities of food items basing on the “go for more” strategy. For example, great apes (Call, 2000; Hanus and Call, 2007), monkeys (Hauser et al., 2000; Uller et al., 2003; Evans et al., 2009), elephants *Elephas maximus* (Irie-Sugimoto et al., 2009; Perdue et al., 2012), domestic dogs *Canis lupus familiaris* (Ward and Smuts, 2007), coyotes *Canis latrans* (Baker et al., 2011), wolves *Canis lupus* (Utrata et al., 2012), sea lions *Otaria flavescens* (Abramson et al., 2011), salamanders *Plethodon cinereus* (Uller et al., 2003), and some other species, when presented with two alternatives each comprised of different numbers of food items, prefer the larger quantity. However, when dealing with uncertainty in the environment, animals cannot simply “go for more.” There are some experimental studies in which members of different species are required to choose between foraging options when risk is generated by variability in the amount of reward or by variability in delay to reward (Kacelnik and Bateson, 1996; Heilbronner et al., 2008; Beran et al., 2009, 2012). In nature animals can face even more risky situations when foraging on prey that differ in their dangerousness. Under such circumstances consumers must be sensitive to the relation between quantity of prey and their potential for injury. For example, in grasshopper mice (*Onychomys spp*; Rowe and Rowe, 2006) and meerkats (*Suricata suricatta*; Thornton, 2008) feeding on neurotoxic scorpions, predators should benefit from assessing the risks posed by prey. We suggest that in such situations animals can distinguish between quantities of dangerous food items and make their decision cautiously basing on risk/reward evaluation. In order to test this hypothesis, we simulated ant-hunting in the striped field mouse *Apodemus agrarius*. This is a common dwelling, agile and exploratory species whose cognitive abilities have, to the best of our knowledge, never been studied. Our intuition about the ability of mice to judge encounters with differing quantities of ants in their feeding territories in order to decide to hunt or flee from numerous biting (but edible) insects is based on our previous studies of relations of *A. agrarius* with red wood ants (*Formica* s.str.) as hunters and mass prey (Panteleeva et al., 2011; Panteleeva and Vygonyailova, 2012). We found that red wood ants and rodents share areas in forest habitats. Within their large feeding territories red wood ants create “black holes” in the habitat, i.e., areas that are highly perilous for other species, both invertebrates and small vertebrates, where intruders can be killed or at least injured. The most risky areas are in the vicinities of anthills and ants' foraging routes (Reznikova and Dorosheva, 2004). Surprisingly, inter-relations between red wood ants and small rodents have not been investigated before. We found that ants have high hedonistic value for striped field mice: in our laboratory experiments rodents always tried to eat as much ants as they could, although they have enough food in their home cages, including proteins (Panteleeva et al., 2011). At the same time, when performing ant hunting, mice face a sophisticated foraging challenge: they cannot simply decide to “go for more” because when their number increase, red wood ants become more and more dangerous for small rodents. We suggested that striped field mice could estimate what is for them the critical level of the dynamic density of ants (individuals per m^2^ per min) in order to hunt comfortably. So, we considered ant-hunting in wild-caught mice as a good model for studying quantity discrimination in an ecological context.

In order to investigate the potential cognitive mechanisms of decision-making in wild-caught field mice, we simulated feeding patches in two experiments in which mice dealt with different quantities of natural stimuli. In the first (preliminary) experiment we investigated to what extent mice were interested in ants as a live prey and how their hunting activity depended on the quantity of edible but rather aggressive insects. We placed mice, both wild-captured and their progeny, one by one into arenas together with ant groups of different quantities, from 10 to 60 individuals, and recorded the behavior of the mice. In the second experiment mice had to choose between different quantities of live prey that served both as food items and as a source of danger. We presented mice with pairs of transparent tunnels with different quantities of live ants inside (5 vs. 15, 5 vs. 30, 10 vs. 30, in different sessions). The tunnels were devised so that mice were able to enter, kill and eat ants there and then leave, whereas ants were locked up. As far as we know, this is the first experimental paradigm based on evaluation of quantity judgments in the context of risk/reward decision making in a simulated ecologically relevant situation where the animals face a trade-off between the hedonistic value of the prey and the danger it presents.

## Experiment 1. predatory behavior of mice toward ants with respect to their quantity

### Subjects and housing

The experiment was conducted in 2009, 2010, and 2012 in the laboratory on striped field mice *A. agrarius*. These mice do not form aggregations during active periods of their annual life cycle, and adults live solitary (Wolff and Sherman, 2007). We used 25 striped field mice (12 females and 13 males), from which 4 (2 males and 2 females) were captured in a mixed-pine forest near Novosibirsk, and 20 were born in the laboratory, being progeny of the wild-caught mice. These “naïve” mice were from 2 to 12 months of age when they were tested. All animals were housed singly in clear plastic cages (40 × 30 × 20 cm) that contained cotton nesting material. Laboratory-born mice were weaned between 25 and 35 days of age, housed with littermates until 40 days of age, and thereafter housed singly in cages. All mice were fed each day with mixed seeds, fruits, and dried shrimps, and they had *ad libitum* access to water. When they were taken into experimental set-ups, mice always had enough food in their home feeders, so they were not hungry. However, as it has been revealed earlier (Panteleeva et al., 2011), ants are rather attractive for them as a prey. Animals voluntarily participated in experiments and readily entered the plastic cup that we used in our manipulations with them.

We used red wood ants as live prey: *Formica polyctena* in 2009 and *F. aquilonia* in 2010 and 2012. These are closely related species which even form common two-species nests (Korcsiñska et al., 2010). It is worth noting that red wood ants do not display considerable variability in size, that is, in our experiments mice were presented with live prey items of approximately the same size. Ants were taken from the same forest with mice and were housed in groups of about 1000 individuals in artificial nests on separate arenas (60 × 50 cm), where they received water, carbohydrate and protein food *ad libitum*.

### Procedure

In the first part of the experiment conducted in 2009, we investigated the process of ant hunting in striped field mice. We placed each mouse into a separate round arena (40 cm in diameter, 20 cm high) containing 10 ants. The arena was covered with a transparent lid in order to prevent animals from getting out. Both ants and rodents could freely move in the arena. In each trial a mouse was placed in the arena 2 min after ants. Video recordings were made during 10 min, using a camera Sony Handycam DCR DVD408. Each mouse was tested 3 times, with the interval 3 days between trials. To analyze ethograms from video records, we used the Observer XT 7.0 (version: 7.0.214, Noldus Information Technology). In total, we analyzed 6 h of video by the second, for 9 laboratory-reared and 4 field-caught mice (39 trials).

In the second part of the experiment conducted in 2010 and 2012 we examined mice' predatory behavior toward different quantities of ants. The apparatus and video recording were the same as in the first part, as well as the analysis of ethograms. We tested 20 laboratory-reared and 3 field-caught mice (12 males and 11 females), 3 times each, with different numbers of ants placed in the arena: 10 ants (35 trials in 2012), 20 ants (25 trials in 2010), 30 ants (30 trials in 2010), 40 ants (20 trials in 2010 and 34 trials in 2012), and 60 (29 trials in 2012). Note that numbers of trials are not always multiples of 3 because 28 trials in which mice did not display any activity toward ants were excluded. We tested each animal for 10 min per trial with 10, 20, and 30 ants, 5 min per trial with 40 ants, and 3 min per trial with 60 ants. We used different intervals for different quantities of ants because it could be traumatic for mice to spend more time in the arena with biting ants. Appropriate interval lengths were established in auxiliary trials, in which the trial was stopped when mice displayed distinct signs of discomfort such as jumping, shaking legs, rubbing eyes, and so on. Intervals between trials in this part of the experiment were from 5 to 24 h for each animal, so that mice had sufficient time to rest. In sum, 206 trials were recorded. We examined the number of killed ants, the number of eaten ants, and the details of mice' behavior, including attacks toward insects and signs of dismay (jumps and “freezing” when an animal stayed motionless with its legs and tail hidden and the head ducked). We defined the efficiency of attacks as the proportion of successful attacks (that is, attacks in which the ant was killed) in the total number of attacks toward ants.

In order to establish the correspondence between numbers of ants placed in our arena and the dynamic density of ants in different parts of their feeding territory in nature, we made auxiliary recordings in the field: a wire frame of the same shape and size as the bottom of the arena was placed on plots chosen in different parts of the ant feeding territory, and we counted all ants captured within the boundaries of the frame during 1 min. In total, 71 of such recordings were made. It turned out that 10 ants placed on the arena corresponds to the dynamic density of ants of about 80 individuals per m^2^ per min, which is characteristic for the periphery of an ants' feeding territory. The value of 60 ants placed in the arena corresponds to the dynamic density of ants about 400 individuals per m^2^ per min, which is characteristic for the vicinity of ant routes and anthills (Reznikova and Dorosheva, 2004).

### Results

#### Ant hunting in striped field mice

**Figure 1:**
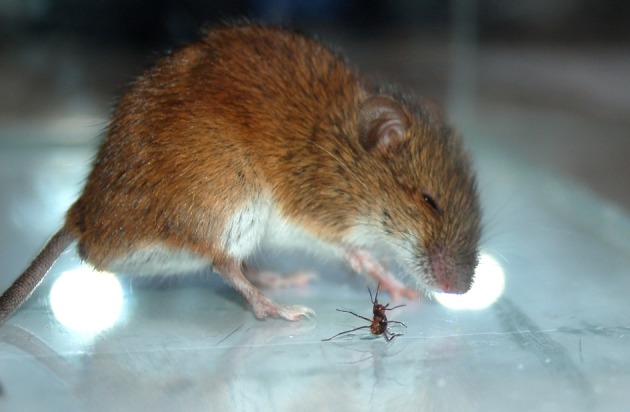
**An ant exhibits the aggressive posture and splash acid toward mouse's eyes**. Photo by Yu. Danilov.

In the first part of the experiment subjects demonstrated predatory behavior toward ants in 33 out of 39 tests. The latency time, that is, the time from the first encounter with an ant until the first attack, lasted from 14 s to 8 min. It is worth noting that ants displayed aggressive behavior toward mice (see: Dorosheva et al., 2011 for a detailed description of signs of aggression in ants): they exhibited aggressive postures, bit rodents on the legs and splashed acid toward their eyes (Figure 1). Mice displayed agitation when damaged by ants, such as twitching, jumping, and rubbing their eyes, and they contacted each insect many times before making their final attack. We recorded 3.48 ± 0.95 contacts with ants per minute which included orienting the body to face the ants, touching insects with the nose, and probing bites that did not end with killing. Mice made a final attack by quickly getting a better grip with their teeth, and then killed and ate the ant holding it in their paws. Rodents thoroughly collected all fallen fragments of insects including legs and even antennae, and ate them. Mice killed 0.37 ± 0.03 ants per minute; thus, their hunting appeared to be quite active, taking into account that they had to cope with the biting prey.

#### Reactions of striped field mice to different quantities of ants

**Figure 2:**
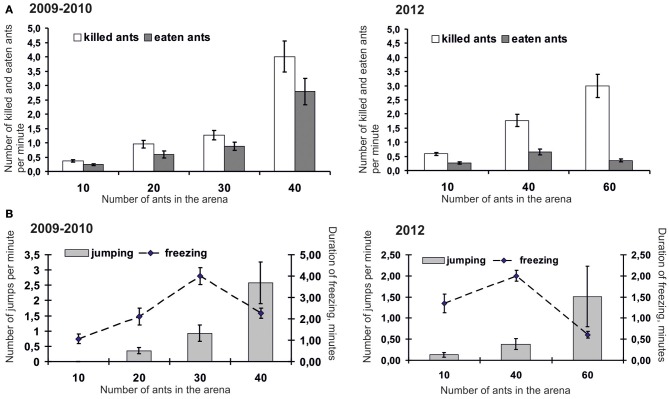
**Number of ants killed and eaten by mice (A), and number of signs of dismay in mice (B) placed in the arena together with different number of ants (Mean ± SE).** Note for the two left graphs: data with 10 ants were obtained in 2009, and the rest were obtained in 2010.

Being presented with different quantities of ants in arenas, mice actively hunted until no ants remained. The number of killed and eaten ants increased with the number of insects (from 10 to 40) placed in the arena (Figure 2). Mice killed up to 4.0 ± 0.50 ants per minute. The efficiency of attacks, that is, the proportion between killed and attacked insects, increased with the number of ants placed in the arena. In 2009–2010 the efficiency of attacks was 11.44 ± 1.34% in trials with 10 ants, 16.83 ± 2.35%, 25.25 ± 3.58%, and 36.26 ± 3.60 in trials with 20, 30, and 40 ants correspondingly. In 2012 the efficiency of attacks was 6.14 ± 0.57%, 17.86 ± 1.80%, and 21.92 ± 2.32 in trials with 10, 40, and 60 ants correspondingly. However, successfully attacking and killing ants did not always lead to mice eating them. Figure 2 shows that when finding themselves in the arena with 30, 40, and 60 ants, mice killed significantly more ants than they were able to eat. It possibly means that mice kill ants in self defence in situations when there are too many aggressive insects around. Indeed, it can hardly be considered comfortable hunting when ants' number in the arena increases up to 40 and 60, and thus becomes comparable with their number in the vicinity of foraging routes and ant-hills in nature. Mice suffered from bites and displayed more and more signs of discomfort such as jumping and freezing; they sharply jumped trying to shake ants off their legs. The number of signs of discomfort significantly increased with the increase in the number of ants placed in the arena. When 40 ants were placed in the arena, mice mainly switched from jumping to freezing (Figure 2). Only when they had brought the number of ants down to an acceptable level were mice able to start collecting killed insects and eat them. Surprisingly, they were able to eat up to all 40 ants during 5 min trials. These data enabled us to conclude that, although ants' attractiveness as prey is rather stable for mice, they have to choose feeding patches with relatively small quantities of active ants in order not to suffer from their bites too much. The next experiment (“Experiment 2”) was aimed at examining whether rodents can use quantity information to make a decision where to hunt ants.

## Experiment 2. distinguishing between quantities of aggressive ants: hunt or flee?

### Subjects and housing

The experiment was conducted in 2011. We used 16 striped field mice (9 males and 7 females), of 4–15 months of age, that were first and second generation descendants of those captured in a mixed-pine forest near Novosibirsk. Mice were housed and fed as it was described in the section “Experiment 1.” As before, animals voluntarily participated in experiments and readily entered the plastic cup that we used in our manipulations with them.

### Apparatus, stimuli, and procedure

**Figure 3:**
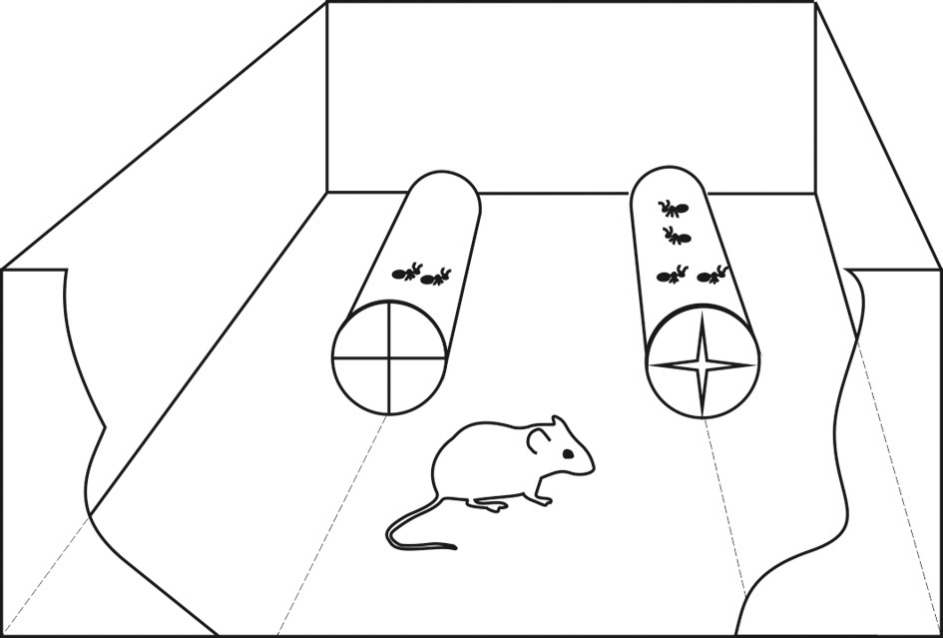
**Experimental setting used: tunnels contain different number of live ants.** It is shown in the right tunnel how crosswise slits allow a mouse to pass in and out by applying its own weight. Note that in reality mice had to choose between 5 and 15 ants (and so on, see the text), and never between 2 and 4.

In order to simulate “feeding patches” with different dynamic densities of active ants, we recycled transparent plastic 0.3 L water bottles in order to make tunnels containing different quantities of live ants that were actively moving inside. These tunnels were presented in such a way that mice could enter them from the bottom side; the bottom was cut off and replaced by an elastic plastic lid with crosswise narrow slits that allowed a mouse to pass in and out by applying its own weight, and did not allow ants to either enter or leave, as they were lightweight and could not press through narrow slits in the elastic lid. Mice were tested one by one in a rectangular container (25 × 35 cm, and 21 cm high) with two tunnels attached to its sides (Figure 3). So, mice were able to enter, kill and eat ants in the tunnel, and then leave, whereas ants were locked up. The subject could see and compare the contents of the two tunnels at the same time. We relied on free hunting behavior of mice that had no previous training history of ant hunting in the containers.

Before being tested with ants, each mouse passed through the training phase of the experiment, being presented with a small piece of cheese inside each tunnel. At this stage there were no ants inside the tunnels. The training phase lasted for each subject until it began to inspect tunnels voluntarily immediately after finding itself in a center of the experimental container. It took usually not more than three training trials for each animal, and after that all of them actively chose containers with ants inside.

During the main course of the experiment animals could freely move in the experimental container (10 min per trial). In order to avoid positional learning (left or right), we changed positions of the tunnels after each trial, so the animals could rely only on the different quantity of ants inside the tunnels. Both tunnels were kept stationary during each trial. As the main characteristic of mice' behavior in the artificial “feeding patches” we recorded all choices of tunnels by each animal during each 10-min trial. We considered a response to be the choice of a tunnel if an animal entered the tunnel completely (including its tail). As supplementary characteristics, we also considered the time duration spent by an animal inside a tunnel, and, separately, all cases when a mouse touched the entrance with its nose or front paws. We considered touching of the entrance as a sign of exploratory activity of mice toward the tunnels.

In different conditions the tunnels contained different numbers of live ants: condition 1: 5 vs. 15 (14 mice were tested); condition 2: 5 vs. 30 (16 mice were tested); condition 3: 10 vs. 30 (14 mice). It is worth noting that, although numbers of ants placed in the container in several cases were the same as in the open arena (for example, 10 and 30), situations differed considerably. Ants were locked within a small volume of 0.3 L where they could move freely in 3-dimensional space, so, when entering a bottle containing ants, a mouse must have felt less comfortable than in the open arena. Each mouse was tested three times with 2 days interval between the trials, and we summarized the data obtained in three trials. The total number of trials was 132, totaling 22 h of duration.

### Statistical analysis

As the main characteristic of mice' behavior in simulated feeding patches, we compared the number of choices the mice made between the two containers during trials. To test whether the mice' choices for one of the tunnels deviated from the chance level we applied Pearson's chi-squared test (χ^2^). To test whether the mice' exploratory activity was higher toward one of the tunnels, we compared numbers of mice' contacts with the lids of the tunnels applying Pearson's chi-squared test (χ^2^). To test whether the mice spent significantly more time in one of the tunnels during trials, we applied the Wilcoxon signed-rank test.

### Results

**Figure 4:**
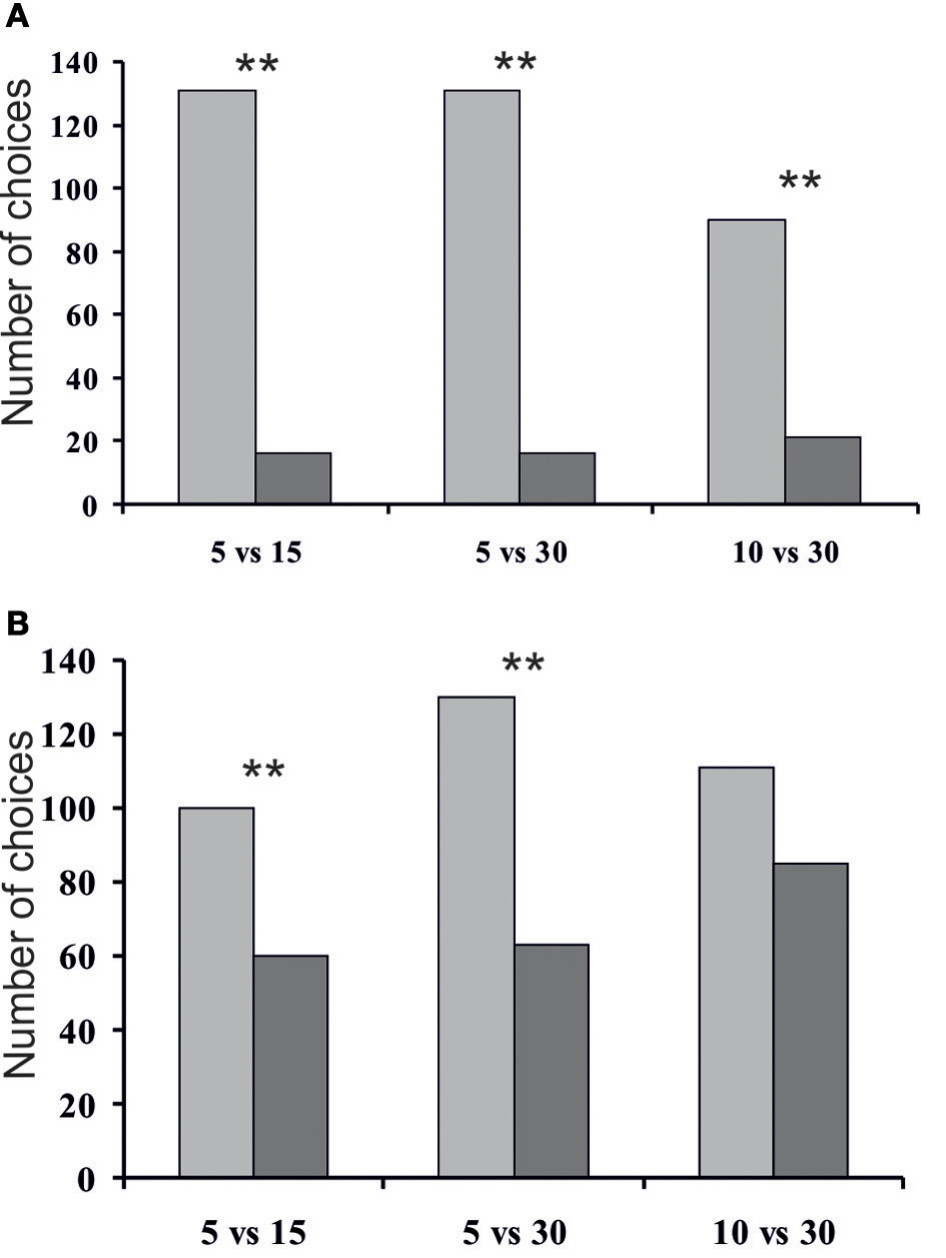
Total number of choices of the tunnels by mice (A) and total number of all cases when mice touched the entrance of the tunnels (B) (^**^P < 0.01 Pearson's chi-squared test).

We compared tunnel exploration by rodents across conditions. Striped field mice appeared to choose significantly more frequently the tunnels containing fewer ants (Figure 4). In condition 1 (5 vs. 15 ants) all mice in sum chose the tunnel containing 5 ants 131 times, whereas the tunnel with 15 ants they chose 16 times (χ^2^ = 89.97, *P* < 0.01). In condition 2 (5 vs. 30 ants) mice chose the tunnel containing 5 ants 131 times, and the tunnel with 30 ants they chose 16 times (χ^2^ = 89.97, *P* < 0.01). In condition 3 (10 vs. 30 ants) the mice chose the tunnel containing 10 ants 90 times, whereas the tunnel with 30 ants they chose 21 times (χ^2^ = 42.89, *P* < 0.01). Taking into account that we tested 16 mice altogether, these proportions mean that, on average, during the first two sessions mice chose the tunnel containing more ants only once, and the number of “go for more” choices during the third session was 1.7 per mouse. Numbers of mice' contacts with the lids significantly differed in conditions 1 and 2 between the smaller quantity and the larger quantity (χ^2^ = 10.00, *P* < 0.01 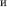 χ^2^ = 32.26, *P* < 0.01, correspondingly). It is important to note that in all three conditions the mice spent significantly more time in the tunnels containing fewer ants (Wilcoxon signed-rank test: session B1: *T* = 0.00, *Z* = 5.37, *P* = 0.00; session B2: *T* = 3.00, *Z* = 5.11, *P* = 0.00; session B3: *T* = 1.00, *Z* = 4.76, *P* = 0.00). During condition 1 the mice spent in total 65.65 min in the tunnel containing 5 ants, and 0.95 min in the tunnel with 15 ants; during session 2 and 3 these values were correspondingly 65.87 vs. 3.83 min, and 39.05 vs. 1.33 min.

Behaviorally, mice caught ants in the tunnel that contained less ants, killed and ate the insects, then quietly left the tunnel and after a short time went back to hunt again. In contrast, they jumped out of the tunnel containing many ants, and rarely visited it again. In either case, these behaviors were independent of the position of the tunnel (right or left).

## Discussion

We developed a naturalistic test of quantity judgments in striped field mice basing on their free hunting behavior, that is, on cognitive abilities that are spontaneously present in the species. Instead of working, as is typically the case, with laboratory strains of rodents (Capaldi and Miller, 1988; Janus et al., 2009), we tested wild-caught striped field mice and their progeny in situations nearest to their vital environmental problems, that is, in simulated feeding patches. To test the hypothesis about the ability of mice to apply the “go for less” strategy in order to ensure comfortable hunting of dangerous prey, we presented mice with a spontaneous choice task in which they were forced to choose between different amounts of ants.

It is worth noting that this is the first study of ant hunting in Muridae, even though insect predation has been described in this family. While nearly every rodent species is to some degree an omnivore (Landry, 1970), the degree of carnivory has been, as far as we know, poorly estimated for Muridae. It is known that striped field mice, although they eat mainly seeds and plants, include a great deal of insects in their diet (Babiñska-Werka, 1981); however, details of insect hunting were not studied in this species. In our previous studies (Panteleeva et al., 2011) we revealed that red wood ants are always attractive as a prey even for replete rodents. The reasons are not entirely clear yet. The high hedonistic value of ants makes mice not only catch and eat them but also collect and eat all fragments of those insects including legs and even antennae. This can perhaps be explained by the high concentration of glucose in their bodies and on their covers (see: Jilková et al., 2012), as well as by the accumulation of certain microelements by them (see: Frouz and Jilková, 2008) as well as proteins. That striped field mice demonstrate stable and active predatory behavior toward aggressive (but edible) red wood ants enabled us to study quantity judgments in the context of risk/reward decision making.

In “Experiment 1” we examined mice' reactions toward different quantities of ants in order to reveal how many ants a mouse can catch without suffering from bites too much. In our study both field-caught and naïve mice displayed rather skilled predatory attacks, and their efficiency of hunting was comparable with that of specialized predators. At the same time, ant hunting appeared to be risky for striped field mice, as they suffered from bites and displayed more uncomfortable reactions when they encountered more ants as opposed to less ants. In our experiments we observed behaviors, including turning to face the ants, touching insects with the nose, and probing bites that did not end with killing, each of which can be considered an element of risk assessment in striped field mice, analogous to those described in grasshopper mice toward dangerous prey (Rowe and Rowe, 2006). The limit of dynamic density (individuals per m^2^ per min) of ants that allows mice to hunt comfortably appeared to be about 80 individuals per m^2^ per min which corresponds to the level of dynamic density in peripheral parts of ants' feeding territories in nature, that is, far away from ant-hills and foraging routes. In sum, ant hunting can serve as a good model for investigating cognitive mechanisms of risk/reward decision making in small rodents.

In “Experiment 2” mice had to choose between 5 vs. 15, 5 vs. 30, and 10 vs. 30 ants placed in two transparent tunnels. The subject could see and compare the content of the two tunnels at the same time. Animals could freely enter the tunnels and hunt there, and so ants served both as food items and as a source of danger. In this situation striped field mice displayed the clear tendency to “go for less” in all three trial types, thus displaying the capacity for distinguishing more from less in order to ensure comfortable hunting. Additional experiments should be conducted to examine what level of accuracy animals can achieve when distinguishing between tunnels containing different quantities of ants, and when they might “go for more” instead of “go for less.” It is also important to note that based on the results of the experiment conducted, we cannot determine the preferred sensory modality used by striped field mice to make their decision. In our experiments mice could estimate quantities of moving visual objects, but they also could use the amount of smell and (or) the patter of ant feet within tunnels as cues. Additional experiments are needed with non-transparent tunnels in which ants would be invisible for mice. However, even if mice in our experiment use cues of different modalities, our results show that they can estimate proportions of edible but dangerous objects and make the decision to hunt or flee based on distinguishing between quantities, possibly evaluating not only moving visual objects but also smells and sounds. These results are ecologically relevant as they reflect situations and challenges faced by free-living small rodents when they have to estimate the frequency of encounters with dangerous prey. It would be interesting to go deeper in studying quantity judgments in “wild” rodent species and test their ability to distinguish between numbers or other quantities of arbitrary stimuli.

### Conflict of interest statement

The authors declare that the research was conducted in the absence of any commercial or financial relationships that could be construed as a potential conflict of interest.
